# Calcium Sulfate Dihydrate With Titanium Scaffold in Conservative Management of a Multicystic Ameloblastoma: A Case Report

**DOI:** 10.7759/cureus.27050

**Published:** 2022-07-20

**Authors:** Deepankar Shukla, Nitin D Bhola, Krutarth Kshirsagar, Pooja Agrawal, Mayur B Wanjari

**Affiliations:** 1 Oral and Maxillofacial Surgery, Sharad Pawar Dental College and Hospital, Datta Meghe Institute of Medical Sciences (Deemed to be University), Wardha, IND; 2 Research and Development, Jawaharlal Nehru Medical College, Datta Meghe Institute of Medical Sciences (Deemed to be University), Wardha, IND

**Keywords:** curettage, enucleation, mandible, titanium mesh, calcium sulfate, ameloblastoma

## Abstract

Ameloblastoma is an odontogenic tumor that is locally invasive and epithelial in origin and accounts for 1% of all jaw tumors and cysts. The body and ramus region of the mandible is where it is most commonly found in the third and fourth decades of life usually. Multicystic ameloblastoma is thought to be the most aggressive kind of ameloblastoma with a high recurrence rate. Surgical treatments are aggressive in nature like marginal or segmental resection have been practiced but conservative managements such as decompression, enucleation, or curettage are also being employed. This case report aimed to demonstrate the conservative management of multicystic ameloblastoma presented in the second decade of life and analyze the treatment outcomes for the same.

## Introduction

Ameloblastoma is the most frequent benign odontogenic tumor. It is usually a painless, slowly growing, locally aggressive tumor that causes cortical bone enlargement, perforation of the lingual or buccal cortical plates, and soft-tissue infiltration. It occurs most commonly in the third and fourth decades of life. The proportion of mandible to maxilla range from 80-20% to 99-1%. Ameloblastoma can appear as unilocular or multilocular corticated radiolucency on a conventional radiograph, with the bone septae forming a honeycomb or soap bubble pattern, or a tennis racket pattern [1]. When treating ameloblastoma, the difficulty in obtaining complete excision and reconstruction of the defect when the tumor is substantial. Depending on the size and form of the lesion, it is treated by enucleation, curettage, or surgical excision. Recurrence rates range from 17.7% after en bloc resection to 34.7% with conservative therapy [2]. To prevent local recurrence, wide resections with a safety margin of healthy bone were preferable.

## Case presentation

**Figure 1:**
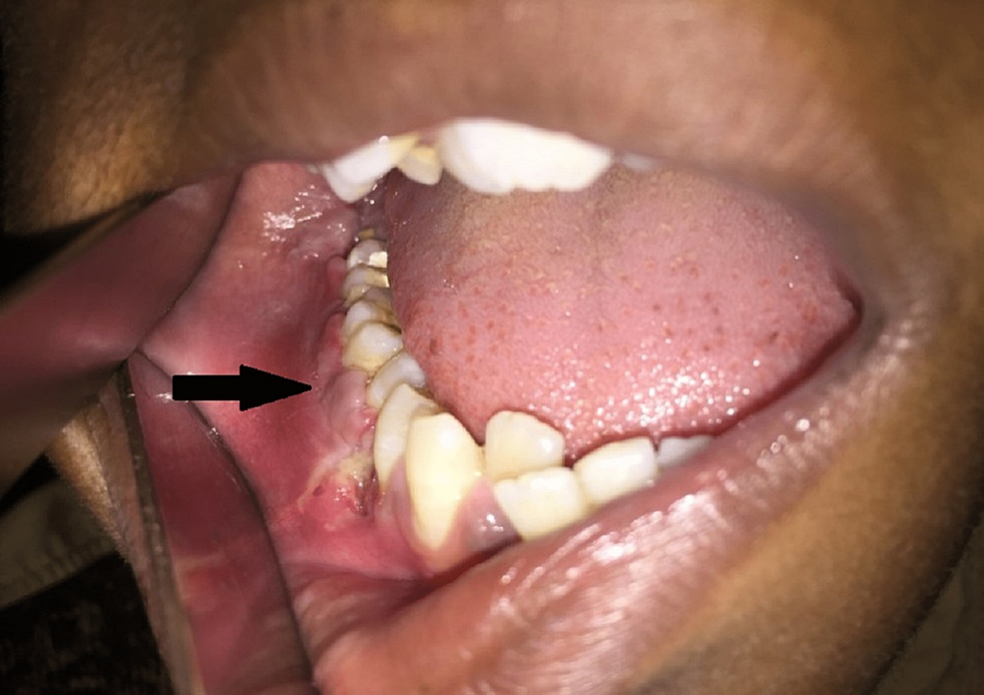
Intra-oral image showing swelling in the lower right gingivobuccal sulcus region

A 17-year-old male, with no medical history, presented with swelling on the right side of the face for the past one and half years. The swelling was initially small and steadily increased until it reached its current size. There was a history of dull aching, and intermittent, and localized pain with no aggravating or relieving causes. There was a history of pus discharge for one month approximately. On examination, there was the presence of a solitary ill-defined diffuse swelling over the right lower third of the face, measuring 5 x 3 cm and extending superoinferiorly from 1 cm below the ala-tragal line of the lower border of the mandible, and mediolaterally from 1 cm posterior to the corner of the mouth to the angle of the mandible. The surface was smooth and the skin overlying the swelling was shiny and normal in color with no signs of any secondary changes seen (Figure 1). On palpation, the swelling was tender and firm. Intra-orally, an ill-defined swelling in the right lower posterior buccal vestibule extending anteroposteriorly from 43-46, with a smooth surface and overlying mucosa was stretched and similar in color to its surrounding mucosa. There was buccal and lingual cortical plate enlargement and it was tender to touch and firm in consistency.

**Figure 2:**
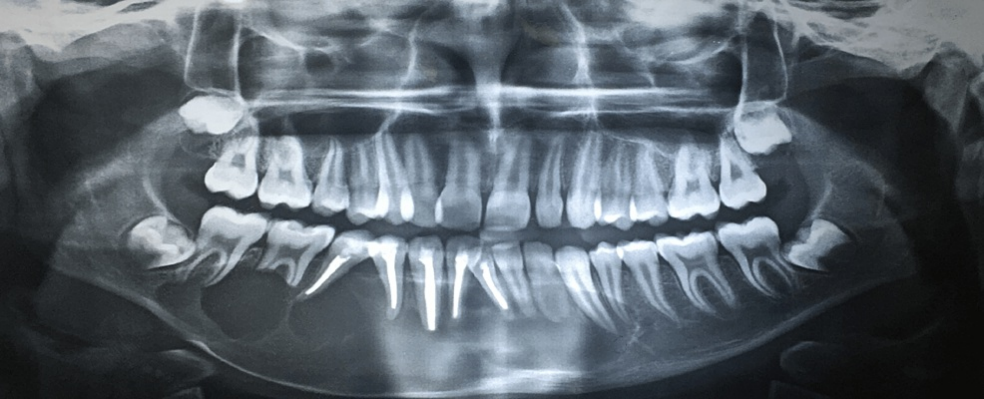
Orthopantomogram showing multicystic radiolucencies in mandibular body-ramus region

Based on clinical data, a tentative diagnosis of the benign mandibular tumor was made. Ameloblastoma was assumed to be the first on the differential diagnosis list as it is the most prevalent tumor in the mandibular ramus region. An orthopantomogram was done which showed multi-cystic radiolucencies in the molar-ramus region causing bone and root resorption of the involved teeth (Figure 2). Further, a bone biopsy was performed and the specimen was subjected to histopathological examination suggestive of follicular ameloblastoma.

**Figure 3:**
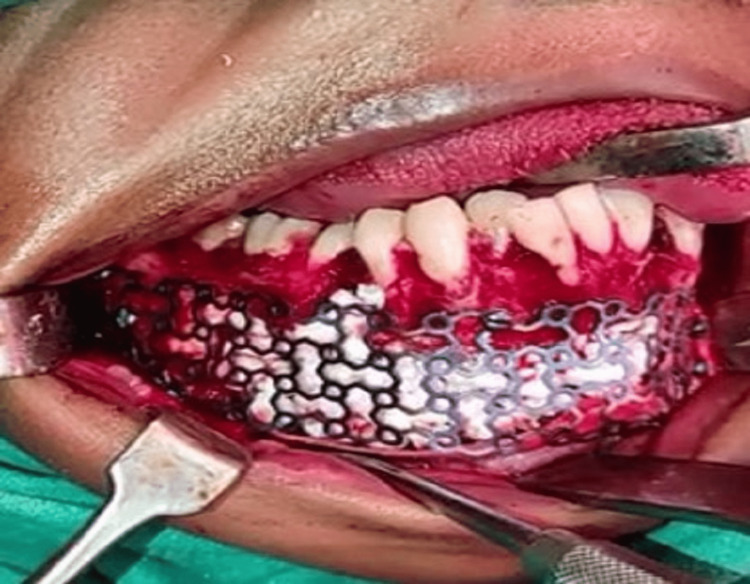
Reconstruction with titanium mesh and calcium sulfate dihydrate bone substitutes

The patient was then planned for surgery after all preoperative investigations. Considering the age of the patient, all the teeth involved were endodontically treated and surgical enucleation, curettage, and peripheral osteotomy of the right mandibular body region followed by reconstruction with titanium mesh, a scaffold for calcium sulfate dihydrate bone graft material were done. Under all aseptic conditions, a crevicular incision was given from 36-47. Enucleation and curettage of the right mandibular body were done and the cystic lining was removed and sent for histopathological examination. Chemical cauterization is done using a modified Carnoy's solution. Access opening was done before the OT with respect to 34-47 tooth numbers and root canal opening was done with 46. Reconstruction with titanium mesh done and fixed by three screws of 2 x 8 mm and two screws of 2 x 6 mm each along with beads of bone graft substitute. In the mixing dish, 240 mg of tobramycin liquid was combined with 5 cc of calcium sulfate. For 30 seconds, the ingredients were mixed till they reached the “doughy” stage. The beads were filled in the surgical defect after drying (Figure 3).

**Figure 4:**
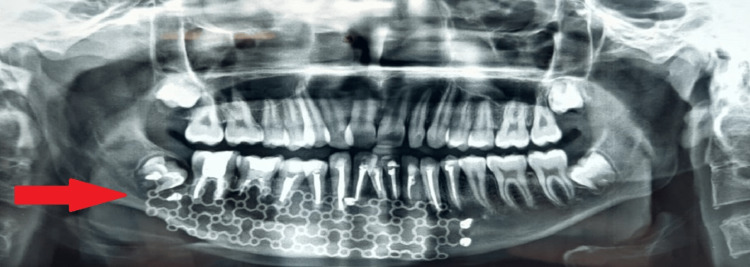
Post-operative orthopantomogram after three months of surgery

Following surgery, the patient was given antibiotics and painkillers. Post-operative instructions were given and regular follow-up was advised. The patient was evaluated clinically and radiographically initially every 10 days and then every three months (Figure 4).

## Discussion

On a clinical basis, ameloblastomas are of three types - solid and multicystic, unicystic, and peripheral ameloblastomas [3]. Swelling and asymmetrical face are the most typical presenting symptoms, with pain being an uncommon sign. Small lesions are sometimes discovered more frequently during routine radiographic screening examinations or as a result of the tumor’s local effects, such as tooth mobility, occlusal alterations, and failure of tooth eruption.

Treatment of ameloblastoma can be conservative or aggressive. Violent curettage of the bone after enucleation should be avoided because it may implant ameloblastoma foci deeper into the bone. Chemical cauterization with Carnoy's is also recommended due to the high chance of recurrence. Because the cystic wall contains islands of ameloblastoma tumor cells, penetration into the surrounding cancellous bone is possible [2]. Late recurrence after treatment is common, with an average recurrence interval of seven years. According to Maia et al., “recurrence rates for resection were 30.5% for enucleation alone, 16% for enucleation followed by Carnoy's solution application, and 18% for marsupialization followed by enucleation” [4].

Depending on the size and location of the lesion, several grafts can be used for reconstruction purposes. Autogenous grafts are considered a gold standard. However, donor site morbidity, restricted availability, and higher surgical time are all disadvantages. Allografts overcome the limitations of autografts but the possibility of viral illness transfer is still present. Xenografts are generated from a genetically different species than the host. Its disadvantage is that it can spread illness. Alloplast has features like osteoconduction and osseointegration and is thereby more popular. Some examples of alloplasts are bioactive glass, glass ionomers, calcium sulfate, calcium phosphates, tri-calcium phosphate, and synthetic hydroxyapatite [5].

Calcium sulfate assisted in gradual bone regrowth. Calcium sulfate is bioresorbable and is available in hemihydrate form. It allows movement of water out of the cells with the accumulation of fluid, and wound drainage and does not cause allergic reactions. It can be assessed radiographically as it is radio-opaque. Hence regular follow-up and radiographic analysis are necessary [6].

## Conclusions

To avoid delay in bone growth and unnecessary pathologic fracture, the treatment approach for a large cystic defect must include prompt bone graft insertion. Also, along with the bone graft material, the insertion of a scaffold to hold the material improvises the result and provides a rigid framework. Calcium sulfate dihydrate along with the insertion of titanium mesh has been shown to be effective in regenerating bone in cystic defects and provides rigid support with few complications in a short period of time as seen in this case.
